# Frequência e fatores relacionados ao índice tornozelo-braquial aberrante em diabéticos

**DOI:** 10.1590/1677-5449.009316

**Published:** 2016

**Authors:** Ana Luisa Guimarães Siqueira de Araújo, Cícero Fidelis, Vanessa Prado dos Santos, José Siqueira de Araújo, Jacy Andrade, Marco Antônio Vasconcelos Rêgo

**Affiliations:** 1 Escola Bahiana de Medicina e Saúde Pública – EBMSP, Faculdade de Medicina, Salvador, BA, Brasil.; 2 Universidade Federal da Bahia – UFBA, Faculdade de Medicina da Bahia, Salvador, BA, Brasil.; 3 Universidade Federal da Bahia – UFBA, Instituto de Humanidades Artes e Ciências Professor Milton Santos – IHAC, Salvador, BA, Brasil.

**Keywords:** índice tornozelo-braquial, diabetes melito, angiopatia, complicações do diabetes

## Abstract

**Contexto:**

O índice tornozelo-braquial (ITB) é um exame de rastreamento da doença arterial obstrutiva periférica, sendo também utilizado para avaliar o risco cardiovascular. Em diabéticos, a interpretação do exame é difícil pela possibilidade de índice aberrante devido à calcificação da camada média arterial.

**Objetivo:**

Encontrar a frequência de ITB aberrante em diabéticos e verificar sua associação com variáveis sociodemográficas.

**Métodos:**

Estudo descritivo com entrevista e aferição de ITB de 309 pacientes diabéticos tipo 2, acompanhados no centro de referência Centro de Diabetes e Endocrinologia da Bahia (CEDEBA), Salvador, BA, Brasil. Foi estudada a frequência e a relação entre o ITB aberrante e variáveis sociodemográficas, como sexo, idade e renda familiar. Utilizou-se um ponto de corte para ITB aberrante de 1,3. As variáveis contínuas foram dicotomizadas. Para a análise estatística, utilizou-se o teste do qui-quadrado, considerando significante um p ≤ 0,05.

**Resultados:**

Entre os 309 pacientes entrevistados, 65% eram mulheres, 26% haviam cursado ensino médio completo e 77% tinham renda familiar igual ou menor que três salários mínimos. A frequência de ITB aberrante ≥ 1,3 foi 16,5%. Não foram encontradas correlações estatisticamente significantes nas análises bivariadas entre o ITB aberrante (≥ 1,3) e as variáveis sociodemográficas estudadas (sexo, idade, tempo de duração de diabetes melito, renda familiar e escolaridade).

**Conclusões:**

A frequência de ITB aberrante entre diabéticos foi de 16,5%. Não encontramos correlação entre as variáveis sociodemográficas (sexo, idade, tempo de DM, escolaridade e renda familiar) e a ocorrência de ITB aberrante.

## INTRODUÇÃO

O índice tornozelo-braquial (ITB) é um exame de rastreamento diagnóstico não invasivo e de boa sensibilidade e especificidade para detecção de doença arterial obstrutiva periférica (DAOP)^1^. O limite superior da normalidade do ITB ainda é controverso na literatura. Foram propostos diversos valores para esse limite, com autores sugerindo valores entre 1,15 e 1,3, e outros estudos de risco cardiovascular sugerindo valores acima de 1,4^2^^-^ 5.

Inicialmente, apenas índices abaixo de 0,9 eram preditivos de doença cardiovascular, porém novos estudos concluíram que índices superiores a 1,4 também implicavam maior mortalidade cardiovascular^4^^,^ 5. Os estudos sugerem que índices elevados em pacientes que apresentam fatores de risco para DAOP, tais como tabagismo, dislipidemia, diabetes melito (DM) e idade avançada, representam risco elevado de doença cardiovascular, com diferentes valores de referência para a avaliação dos índices aberrantes^3^^,^ 4 ^,^ 6 ^,^ 7.

No caso específico dos pacientes diabéticos, o ITB pode não avaliar adequadamente a circulação periférica, pois, nesses doentes, existe uma elevada ocorrência de ITB aberrante, estimada em cerca de 21%^4^^,^ 5. Esse fenômeno é secundário à calcificação da camada média arterial, mais prevalente entre diabéticos^8^. O ITB falsamente elevado em pacientes diabéticos pode dificultar a avaliação da aterosclerose periférica e reduzir sua confiabilidade^9^^-^ 11.

Entendendo a importância do ITB como método diagnóstico não invasivo na DAOP e seu papel na avaliação do risco cardiovascular, o objetivo deste estudo é verificar a frequência e os possíveis fatores associados ao ITB aberrante, considerando o valor de 1,3 em pacientes diabéticos.

## MÉTODOS

Realizou-se um estudo descritivo em um centro de referência para tratamento do DM no estado da Bahia, o Centro de Diabetes e Endocrinologia da Bahia (CEDEBA). Foram incluídos 309 pacientes portadores de DM tipo 2 acompanhados no ambulatório do CEDEBA, os quais foram atendidos de forma consecutiva. Os pacientes foram escolhidos de forma aleatória simples por inclusão consecutiva. O ITB foi aferido por um único examinador.

Incluímos pacientes portadores de DM tipo 2, sem úlcera ativa no pé e que concordaram em participar do estudo, assinando o termo de consentimento livre e esclarecido. Foram excluídos pacientes de acordo com os seguintes critérios: portadores de DM tipo 1; pacientes com ITB ≤ 0,8 mmHg (valores indicativos de isquemia); pacientes com antecedente de amputação maior (acima do nível do médio tarso) unilateral ou bilateral de membros inferiores; menores de 18 anos; portadores de doença mental; gestantes e presidiários.

Consideramos o ponto de corte 1,3 para a análise da frequência e dos fatores associados ao ITB aberrante. Foram avaliadas, em relação à ocorrência do ITB aberrante, as variáveis: sexo, idade, tempo de diagnóstico do DM, escolaridade e renda familiar.

A variável tempo de DM foi definida como o intervalo de tempo, em anos, decorrido entre o ano em que o paciente recebeu diagnóstico da doença através de exame complementar específico e o momento da entrevista deste estudo.

Para a medida do ITB, verificou-se a pressão sistólica dos membros superiores (MMSS) e inferiores (MMII) com um tensiômetro convencional, substituindo o estetoscópio tradicional por um equipamento tipo Doppler vascular portátil de 10 mhz e um tensiômetro convencional aferido e revisado^12^.

A técnica da medida do ITB foi o posicionamento do manguito do tensiômetro de forma habitual nos MMSS (acima da dobra do cotovelo) e logo acima dos maléolos (tornozelo) nos MMII com o paciente na posição supina; posicionamento da ponta do transdutor do ultrassom Doppler na projeção da artéria braquial e das artérias dorsal do pé (pediosa) e tibial posterior; insuflação do manguito do tensiômetro até o som do fluxo sanguíneo se tornar inaudível e, em seguida, desinsuflação até se ouvir o primeiro som do fluxo sanguíneo, que corresponde à pressão sistólica máxima; e consideração do ITB dos MMII direito e esquerdo como o valor da maior pressão do tornozelo direito e do esquerdo, respectivamente, dividido pela maior pressão dos MMSS.

Os dados foram analisados no estudo por meio do programa Statistical Package for the Social Sciences – SPSS®, versão 20.0. O banco de dados com os 309 casos foi analisado para o cálculo da frequência de ITB aberrante (≥ 1,3). Foram feitas as análises para caracterização da população, em variáveis contínuas e dicotomizadas. Foi realizada uma análise bivariada (teste do qui-quadrado) correlacionando o ITB aberrante com: idade (mais jovens *vs* mais velhos), sendo 56 anos o ponto de corte adotado; tempo de DM (menor que 10 anos e maior ou igual a 10 anos); escolaridade (até ensino médio incompleto e ensino médio completo ou superior); sexo (masculino ou feminino); e renda familiar (menor ou maior igual a três salários mínimos). A observação do comportamento na análise contínua da distribuição das variáveis indicou os valores para a dicotomização das mesmas. Com relação às variáveis dicotomizadas, o ponto de corte para a idade em 56 anos foi escolhido com base na observação da distribuição etária da população estudada, de modo que 56 anos correspondeu à mediana encontrada. A dicotomização do tempo de DM seguiu o mesmo princípio, constatando-se que a maioria da população possuía mais de 10 anos de doença. Para as demais variáveis (escolaridade e renda familiar), o mesmo critério foi adotado. Após a observação da distribuição desses fatores sociodemográficos na amostra, optou-se por dicotomizá-los nos valores de referência nos quais se encontravam a maioria dos doentes: ensino médio completo ou acima e ensino médio incompleto, para a escolaridade, e maior ou igual a três salários mínimos e menor que três salários mínimos, para a renda familiar. A renda, medida em salários mínimos, teve como vigente na época do estudo o valor de R$ 510,00. Para duas das variáveis estudadas (faixa etária e tempo de duração de DM), utilizamos ainda a estratificação dos doentes em três grupos, para verificar se havia correlação entre as variáveis e a frequência de ITB aberrante, visto que a idade e o DM podem estar implicados na calcificação da camada média arterial^13^.

O estudo seguiu as instruções contidas na Resolução 196/96 do Conselho Nacional de Saúde do Ministério de Saúde. Todos os participantes assinaram o termo de consentimento livre e esclarecido, e a pesquisa foi aprovada pelo Comitê de Ética em Pesquisa do CEDEBA.

## RESULTADOS

Na nossa amostra, que incluiu 309 doentes, houve predominância do gênero feminino (65%). Observou-se que 90,9% dos indivíduos tinham idade entre 40 e 70 anos. Na distribuição etária por grupos, 2,3% dos pacientes tinham 40 anos ou menos; 21,4%, entre 41 e 50 anos; 43%, entre 51 e 60 anos; 26,5%, entre 61 e 70 anos; 5,8%, entre 71 e 80 anos; e 1%, acima de 80 anos. Quase 50% dos indivíduos cursaram o primeiro grau completo, 20,4% tinham o segundo grau incompleto e apenas 8,4% informaram que nunca haviam estudado. Com relação ao tempo de DM, constatou-se que a maioria (68,6%) referia 10 ou mais anos de duração da doença. Na estratificação dos grupos, 13,3% dos pacientes tinham a doença com menos de 5 anos de diagnóstico; 17,5%, com 5 a 10 anos; 22,4%, com 10 a 15 anos; 18,8%, com 15 a 20 anos; e 28%, tinham diagnóstico há 20 anos ou mais. A mediana do tempo de DM foi de 13 anos, com desvio padrão de 8,0046; o valor máximo encontrado foi de 40 anos, e o mínimo, 0,4 anos. A mediana de idade foi de 56 anos, com desvio padrão de 9,172, e valores mínimos e máximos de 26 e 84, respectivamente. A maioria dos indivíduos (77%) tinha renda familiar igual ou menor que três salários mínimos. Considerando a divisão em dois grupos, mais jovens (com idade inferior a 56 anos) e mais velhos (com idade igual ou superior a 56 anos), pouco mais da metade dos doentes (53,1%) enquadrava-se no grupo mais velhos. Quanto ao tempo de DM, a maioria dos pacientes (68,6%) tinha 10 anos ou mais de diagnóstico. A Tabela 1 detalha as características da amostra estudada.

**Tabela 1:** Características dos 309 doentes diabéticos incluídos na amostra e frequência do índice tornozelo-braquial (ITB) aberrante (≥ 1,3).

**Características da amostra (n = 309)**		**n (%)**
Sexo	Masculino	108 (35%)
	Feminino	201 (65%)
Idade	< 56 anos (mais jovens)	145 (46,9%)
	≥ 56 anos (mais velhos)	164 (53,1%)
Escolaridade	Até ensino médio incompleto	226 (73,1%)
	Ensino médio completo ou superior	83 (26,9%)
Renda familiar	≥ 3 salários mínimos	34 (11%)
	< 3 salários mínimos	275 (89%)
Tempo de diagnóstico do diabetes melito	< 10 anos	96 (31,2%)
	≥ 10 anos	212 (68,6%)
ITB ≥ 1,3	Não	258 (83,5%)
	Sim	51 (16,5%)

Considerando o valor ≥ 1,3 como ITB aberrante, observou-se que 16,5%, dos 309 diabéticos apresentaram ITB aberrante (Tabela 1).

A frequência de ITB aberrante foi estudada em relação a duas das variáveis pesquisadas, a faixa etária e o tempo de duração do diabetes, estratificadas em três grupos distintos de doentes. Os resultados detalhados estão na Tabela 2.

**Tabela 2:** Frequência do índice tornozelo-braquial (ITB) aberrante (≥ 1,3) entre os 309 doentes da amostra, estratificados em três grupos segundo idade e tempo de diagnóstico do diabetes.

**Características da amostra quanto a idade e tempo de diabetes (n = 309)**		**Frequência ITB ≥ 1,3** **n (%)**	***Valor de p***
Idade	≤ 60 anos	36 (17,4%)	
	61 a 70 anos	12 (14,8%)	0,77
	> 70 anos	03 (14,3%)	
Tempo de diagnóstico do diabetes melito	< 10 anos	12 (12,5%)	
	10 a 20 anos	23 (18,1%)	0,45
	≥ 20 anos	16 (18,8%)	

Foi realizada uma análise bivariada através do teste do qui-quadrado correlacionando o ITB aberrante (≥ 1,3) com as variáveis sociodemográficas dicotomizadas da amostra (idade, sexo, tempo de DM, escolaridade e renda familiar). Correlacionando essas variáveis com o ITB aberrante (≥ 1,3), não foi encontrada associação estatisticamente significante. Na Tabela 3, encontra-se a análise realizada entre as variáveis dicotomizadas estudadas e a ocorrência do ITB ≥ 1,3 (aberrante).

**Tabela 3:** Análise da correlação entre as variáveis estudadas e a frequência do índice tornozelo-braquial (ITB) aberrante (≥ 1,3) (n = 309 doentes diabéticos).

**Variável**		**ITB < 1,3**	**ITB ≥ 1,3**	***Valor de p***
		**n (%)**	**n (%)**	
Sexo	Masculino	92 (85%)	16 (15%)	0,55
	Feminino	166 (83%)	35 (17%)	
Idade	< 56 anos	121 (83%)	24 (17%)	0,98
	≥ 56 anos	137 (84%)	27 (16%)	
Escolaridade	Até ensino médio incompleto	191 (85%)	35 (15%)	0,42
	Ensino médio completo ou mais	67 (81%)	16 (19%)	
Renda familiar	≥ 3 salários mínimos	27 (79%)	07 (21%)	0,49
	< 3 salários mínimos	231 (84%)	44 (16%)	
Tempo de diagnóstico do diabetes melito	< 10 anos	84 (88%)	12 (12%)	0,19
	≥ 10 anos	173 (82%)	39 (18%)	

## DISCUSSÃO

O ITB é um exame importante para o diagnóstico e prognóstico da DAOP. No entanto, em pacientes portadores de diabetes, ele pode se apresentar falsamente elevado ou aberrante em decorrência da calcificação da camada média arterial, que dificulta ou impede a interrupção do fluxo arterial durante a insuflação do manguito de pressão.

Um dos desafios para a utilização do ITB como fator prognóstico de doença aterosclerótica e cardiovascular é a dificuldade de se definir um ponto de corte ideal para o ITB aberrante, indicativo de calcificação da camada média arterial, que dificulta a compressão pelo manguito. A literatura ainda sugere que, para fins diagnósticos, o valor mais seguro seria a partir de 1,3^2^^-^ 4 ^,^ 6 ^,^ 7 ^,^ 14, pois já seria possível comprovar, e não apenas sugerir, que os pacientes têm calcificação da camada média arterial.

O ITB como exame prognóstico para mortalidade por doença cardiovascular, indicado como fator de risco, é, mais frequentemente, o ITB abaixo de 0,9^4^^,^ 5. No entanto, com relação ao ITB aberrante, um estudo internacional aponta que valores elevados, acima de 1,4, também podem representar maior risco de mortalidade cardiovascular^4^. Publicações nacionais apontam o valor de 1,3 como ponto de corte para o ITB aberrante, quando pode ser considerado fator de risco cardiovascular^15^^,^ 16. Alguns autores sugerem que os processos patológicos se iniciam em torno de 1,15, de modo que o risco cardiovascular pode se tornar relevante a partir desse ponto^3^^,^ 4 ^,^ 8.

No nosso estudo, utilizamos como ponto de corte o valor proposto pela maioria dos estudos; 1,3^2^^,^ 3. A frequência de ITB aberrante (≥ 1,3) na nossa amostra, que só incluiu portadores de DM tipo 2, foi de 16,5%, abaixo da frequência descrita na literatura internacional, que varia em torno de 21%^4^^,^ 5. Moon et al.^13^ realizaram um estudo para avaliar a presença de calcificação da camada média arterial através do exame radiográfico, encontrando essa alteração em 21,2% dos diabéticos e em apenas 5% dos indivíduos não diabéticos. Um estudo brasileiro, que avaliou o ITB de 73 doentes diabéticos com média de idade de 55,7 anos, encontrou que 9,6% deles tinham ITB > 1,4^17^.

Com relação à faixa etária da amostra, a maioria dos pacientes se encontrava acima dos 56 anos, tendo sido encaminhados a um centro de referência ambulatorial para tratamento de diabetes com complicações da doença. Não encontramos associação entre ITB aberrante e a idade dicotomizada em 56 anos; embora os mais jovens tenham apresentado menor frequência de ITB aberrante, a diferença não foi estatisticamente significante. Uma vez que o processo de envelhecimento propicia a calcificação da camada média, e outros autores encontraram a associação da idade com tal achado radiológico^13^, estratificamos os doentes em três faixas etárias distintas. Também não encontramos associação significativa entre os três grupos, de diferentes idades, e a ocorrência de ITB ≥ 1,3. Acreditamos que a não associação entre idade e ITB aberrante, na nossa pesquisa, possa ser atribuída tanto ao tamanho da amostra como também ao fato de que mais de 40% dos doentes tinham idade menor que 56 anos, sendo provavelmente jovens para a instalação do processo de calcificação arterial.

Outra associação testada foi a do tempo de diagnóstico do DM tipo 2 e o ITB aberrante. Seria esperado que os pacientes com maior tempo de duração da doença apresentassem uma maior frequência de ITB aberrante. Os pacientes com tempo de duração do diagnóstico de diabetes acima de 10 anos apresentaram maior prevalência de ITB aberrante, porém sem significância estatística. Estratificamos a mostra em três grupos na busca por essa associação, mas apesar de uma frequência de ITB aberrante ligeiramente maior quanto maior fosse a duração do diabetes, a diferença também não se mostrou estatisticamente significante. Pensamos que, para muitos dos nossos doentes, talvez seja difícil estipular o tempo de duração do diabetes. Muitos brasileiros têm dificuldade de acesso e acompanhamento em serviços de saúde, sendo que o momento do diagnóstico do diabetes se torna impreciso e subjetivo. A descoberta da doença, por vezes, ocorre no advento das suas complicações, e ainda há a dificuldade em determinar o momento exato do diagnóstico. Assim, é difícil obter uma avaliação fidedigna dessas datas.

As avaliações referentes aos outros dados sociodemográficos (renda familiar e escolaridade) relacionados ao ITB aberrante também não demonstraram associação estatisticamente significante. Não encontramos na literatura evidências que demonstrem tais fatores como modificadores da frequência de ITB aberrante, mas estudamos esses fatores pois sabemos das dificuldades que os doentes têm de encontrar informação e acompanhamento adequado, que auxiliem no controle e propiciem uma menor ocorrência de complicações da doença.

Na nossa análise, observamos uma frequência de ITB aberrante ligeiramente maior no sexo feminino, sem diferença significativa entre os sexos. Moon et al.^13^ encontraram associação entre sexo masculino e calcificação da camada média diagnosticada ao exame radiográfico. Esse achado pode se dever ao fato de que a nossa amostra é composta em sua maioria por mulheres. Um estudo brasileiro mostrou que, em pacientes com isquemia crítica, o gênero feminino apresentava maior prevalência de DM e maior extensão da DAOP, com menor número de artérias opacificadas na angiografia da perna^18^.

O nosso trabalho discute um tema importante para o acompanhamento do paciente diabético e avalia características socioeconômicas desse grupo, tendo realizado um exame exequível e de baixo custo (ITB) com potenciais benefícios para essa população. Ele reforça ainda a necessidade da mensuração do ITB na avaliação ambulatorial do doente diabético, pois para além da importância diagnóstica, políticas públicas de saúde podem ser embasadas em dados sedimentados a respeito desse grupo de doentes, que estaria em maior risco cardiovascular. Nossa pesquisa conta com as limitações inerentes a um estudo transversal, que não avaliou, neste momento, outros importantes fatores associados a DM, comorbidades e evolução da doença arterial periférica ou cardiovascular dos doentes.

## CONCLUSÕES

Concluímos que a frequência de ITB aberrante (≥ 1,3) em doentes diabéticos do tipo 2 foi de 16,5%. Na nossa amostra, não encontramos correlação entre sexo, idade, tempo de DM, escolaridade e renda familiar com a ocorrência de ITB aberrante.
