# Supplementary material for: Frequência e fatores relacionados ao índice tornozelo-braquial aberrante em diabéticos
Source: J Vasc Bras. 2016 Jul-Sep;15(3):176–81. [Article in Portuguese] doi: 10.1590/1677-5449.009316 (PMC5829753; doi:10.1590/1677-5449.009316)
Supplement: Translation

# Translation: Frequency and factors associated with high Ankle-Brachial Index in diabetic patients

**Authors:** Ana Luisa Guimarães Siqueira de Araújo, Cícero Fidelis, Vanessa Prado dos Santos, José Siqueira de Araújo, Jacy Andrade, Marco Antônio Vasconcelos Rêgo

**Affiliations:** 1 Escola Bahiana de Medicina e Saúde Pública – EBMSP, Faculdade de Medicina, Salvador, BA, Brazil.; 2 Universidade Federal da Bahia – UFBA, Faculdade de Medicina da Bahia, Salvador, BA, Brazil.; 3 Universidade Federal da Bahia – UFBA, Instituto de Humanidades Artes e Ciências Professor Milton Santos – IHAC, Salvador, BA, Brazil.

**Keywords:** ankle-brachial index, diabetes mellitus, angiopathy, complications of diabetes

## Abstract

**Background:**

The ankle-brachial index (ABI) is a screening test for peripheral arterial occlusive disease and it can also be used to assess cardiovascular risk. However, in diabetics it can be difficult to interpret because the index may be excessively high because of calcification of the arterial tunica media.

**Objective:**

To determine the frequency of high ABI in diabetics and to test for associations with sociodemographic variables.

**Methods:**

This was a descriptive study in which 309 type 2 diabetes patients were interviewed and had their ABI measured. The sample was recruited at a referral center for diabetes and endocrinology (CEDEBA) in Salvador, BA, Brazil. The frequency of excessively high ABI and its relationships with sociodemographic variables such as sex, age and family income were studied. The cutoff point chosen for excessively high ABI was 1.3. Continuous variables were dichotomized. The chi-square test was used for statistical analysis and results with p ≤ 0.05 were considered significant.

**Results:**

A total of 309 patients were interviewed, 65% were women, 26% had graduated from secondary education and 77% had a family income equal to or less than three times the minimum salary. The frequency of excessively high ABI (≥ 1.3) was 16.5%. Bivariate analyses detected no statistically significant correlations between excessively high ABI (≥ 1.3) and the sociodemographic variables studied (sex, age, time since diagnosis of diabetes mellitus, family income and educational level).

**Conclusions:**

The frequency of high ABI among this sample of diabetics was 16.5%. We did not detect correlations between the sociodemographic variables (sex, age, duration of DM, educational level and family income) and high ABI.

## INTRODUCTION

The ankle-brachial index (ABI) is a noninvasive diagnostic screening test with good sensitivity and specificity for detection of peripheral arterial occlusive disease (PAOD).^1^ The upper limit for normal ABI results is still under debate in the literature. Several different cutoff values have been proposed for this limit, with authors suggesting values ranging from 1.15 to 1.3, and other studies of cardiovascular risk suggesting values over 1.4.^2^^-^ 5

Initially, only ABIs below 0.9 were considered predictive of cardiovascular disease, but later studies concluded that ABIs greater than 1.4 were also associated with higher cardiovascular mortality.^4^^,^ 5 Studies suggest that elevated ABI in patients who have risk factors for PAOD such as smoking, dyslipidemia, diabetes mellitus (DM), and advanced age were indicative of elevated risk of cardiovascular disease, with different reference values for evaluation of high indices.^3^^,^ 4 ^,^ 6 ^,^ 7

In the specific case of diabetic patients, ABI may not provide an adequate assessment of peripheral circulation because there is a rate of anomalously high ABI in this group of patients, estimated at around 21%.^4^^,^ 5 This phenomenon is secondary to calcification of the arterial tunica media, which is more prevalent among diabetics.^8^ A falsely elevated ABI in a diabetic patient can make it more difficult to assess peripheral atherosclerosis, reducing the test’s reliability.^9^^-^ 11

Understanding the importance of the ABI as a noninvasive diagnostic method for PAOD and its role for assessment of cardiovascular risk, the objective of this study was to determine the frequency and possible factors associated with high ABI, using a cutoff value of 1.3 with a sample of diabetic patients.

## METHODS

A descriptive study was conducted at a referral center for diabetes and endocrinology (Centro de Diabetes e Endocrinology da Bahia - CEDEBA) in the state of Bahia (Brazil). A total of 309 type 2 diabetes patients who were in outpatients treatment at CEDEBA were recruited consecutively. The patients were selected in a simple random manner by consecutive enrollment. They all had their ABI measured by the same person.

We recruited patients with type 2 DM who had no active foot ulcers and agreed to take part in the study, signing free and informed consent forms. We excluded patients according to the following criteria: type 1 DM patients; patients with ABI ≤ 0.8 mmHg (values indicative of ischemia); patients who had had prior unilateral or bilateral major lower limb amputations (above the level of the midfoot); those less than 18 years old; patients with mental diseases; pregnant women and prison inmates.

We defined a cutoff point of 1.3 for analysis of the frequency of high ABI and factors associated with it. The relationships between high ABI and the following variables were evaluated: sex, age, time since diagnosis of DM, educational level and family income.

The variable duration of DM was defined as the time elapsed (in years), since the year in which the patient’s diagnosis of the disease was confirmed by a specific supplementary test and the date of the interview for this study.

In order to calculate ABI, systolic pressure in upper limbs (UL) and lower limbs (LL) was measured using a conventional blood pressure meter, substituting the traditional stethoscope for a portable 10 mhz Doppler vascular ultrasound device and a conventional blood pressure meter that had been calibrated.^12^

The technique used to measure pressures for ABI was to position the cuff of the blood pressure meter in the normal manner on UL (above the elbow joint) and close above the ankle for the LL, with the patient in a supine position; while positioning the transducer of the Doppler ultrasound device at the projection of the brachial artery and the dorsal arteries of the foot (pedal) and the posterior tibial arteries; before inflating the cuff of the blood pressure meter until the sound of blood flow was no longer audible and then allowing it to deflate until the sound of blood flow was once more audible, which corresponds to maximum systolic pressure; and the highest pressure in right or left LL was divided by the highest pressure in either UL to find the ABI.

Study data were analyzed using the Statistical Package for the Social Sciences, SPSS®, version 20.0. A database was constructed with the results for all 309 cases and then analyzed to calculate the frequencies of high ABI (≥ 1.3). Analyses were also conducted to characterize the population in terms of continuous and dichotomized variables. A bivariate analysis (chi-square test) was performed to calculate correlations between excessively high ABI and the following variables: age (younger vs. older patients), with a cutoff point of 56 years; duration of DM (less than 10 years vs. greater than or equal to 10 years); educational level (less than full secondary education vs. graduated from secondary education and/or higher education); sex (male vs. female); and family income (less than vs. greater than or equal to three times the minimum salary). The values used for dichotomization of variables were chosen on the basis of observations of the behavior of their distribution as continuous variables. With regard to the dichotomized variables, the cutoff point chosen for age (56 years) was based on observation of the age distribution of the study population and 56 years corresponds to the median age. Dichotomization of duration of DM was based on the same principle, since it was found that the majority of the population had had the disease for more than 10 years. The same criterion was adopted for the other variables (educational level and family income). After observation of the distribution of these sociodemographic factors within the sample, it was decided to dichotomize them at reference values that encompassed the majority of patients: graduated from secondary education or higher education vs. not completed secondary education, for educational level; and greater than or equal to three times the minimum salary vs. less than three times the minimum salary for family income. At the time the study was conducted, the minimum salary in force was R$ 510.00 per month. For two of the variables studied (age group and duration of DM) we also stratified the sample into three subsets, to test for correlations between these variables and frequency of high ABI, since age and DM may be involved in calcification of the arterial tunica media.^13^

This study was designed in accordance with the instructions contained in the Brazilian Ministry of Health’s National Health Council resolution 196/96. All participants signed free and informed consent forms and the study was approved by the Research Ethics Committee at CEDEBA.

## RESULTS

The majority of our sample of 309 patients were female (65%). It was observed that 90.9% of the participants were aged from 40 to 70 years old. Age distribution by groups was as follows: 2.3% of the patients were 40 years old or younger; 21.4% were from 41 to 50 years old; 43%, from 51 to 60 years old; 26.5%, from 61 to 70 years old; 5.8%, from 71 to 80 years old; and 1% were more than 80 years old. Almost 50% of them had completed primary school, 20.4% had started but not completed secondary education and 8.4% had not attended school. With regard to duration of DM, it was found that the majority (68.6%) reported being diagnosed 10 years previously or more. When stratified by groups, 13.3% of the patients had had the disease for less than 5 years since diagnosis; 17.5%, for 5 to 10 years; 22.4%, for 10 to 15 years; 18.8%, for 15 to 20 years; and 28% had been diagnosed 20 years or more previously. Median duration of DM was 13 years, with a standard deviation of 8.0046 and a range of 0.4 years to 40 years. Median age was 56 years, with a standard deviation 9.172 and a range of 26 to 84 years. The majority of participants (77%) had a family income equal to or less than three times the minimum salary. When dichotomized into younger (age below 56 years) and older patients (ages greater than or equal to 56 years), a little over half of the participants (53.1%) were in the older subset. With regard to duration of DM, the majority of patients (68.6%) had been diagnosed for 10 years or more. Table 1 lists the characteristics of the study sample.

**Table 1:** Characteristics of the sample of 309 diabetic patients and frequencies of high ankle-brachial index (ABI ≥ 1.3).

**Characteristics of the sample (n = 309)**		**n (%)**
Sex	Male	108 (35%)
	Female	201 (65%)
Age	< 56 years (younger)	145 (46.9%)
	≥ 56 years (older)	164 (53.1%)
Educational level	Up to incomplete secondary education	226 (73.1%)
	Graduated secondary education or attended higher education	83 (26.9%)
Family income	≥ 3 times minimum salary	34 (11%)
	< 3 times minimum salary	275 (89%)
Time since diagnosis of diabetes mellitus	< 10 years	96 (31.2%)
	≥ 10 years	212 (68.6%)
ABI ≥ 1.3	No	258 (83.5%)
	Yes	51 (16.5%)

Taking the value ABI ≥ 1.3 as the cutoff for high ABI, it was found that 16.5% of the 309 diabetics had high ABI (Table 1).

The frequency distribution of high ABI was analyzed in relation to two of the study variables, age group and duration of diabetes, stratified into three distinct subsets of patients. The results are shown in Table 2.

**Table 2:** Frequencies of high ankle-brachial index (ABI ≥ 1.3) among the 309 patients in the sample, stratified into three subsets by age and time since diabetes diagnosis.

**Characteristics of the sample in terms of age and time since diagnosis of diabetes (n = 309)**		**Frequency of ABI ≥ 1.3** **n (%)**	***p-value***
Age	≤ 60 years	36 (17.4%)	
	61 to 70 years	12 (14.8%)	0.77
	> 70 years	03 (14.3%)	
Time since diabetes diagnosis	< 10 years	12 (12.5%)	
	10 to 20 years	23 (18.1%)	0.45
	≥ 20 years	16 (18.8%)	

A bivariate analysis was conducted using the chi-square test to identify correlations between excessively high ABI (≥ 1.3) and the dichotomized sociodemographic variables for the sample (age, sex, time since DM diagnosis, educational level, and family income). No statistically significant associations were detected between any of these variables and excessively high ABI (≥ 1.3). Table 3 lists the results of the analysis of the dichotomized variables studied and high ABI ≥ 1.3.

**Table 3:** Analysis of correlations between variables studied and frequency of high ankle-brachial index (ABI ≥ 1.3) (n = 309 diabetic patients).

**Variable**		**ABI < 1.3**	**ABI ≥ 1.3**	***p-value***
		**n (%)**	**n (%)**	
Sex	Male	92 (85%)	16 (15%)	0.55
	Female	166 (83%)	35 (17%)	
Age	< 56 years	121 (83%)	24 (17%)	0.98
	≥ 56 years	137 (84%)	27 (16%)	
Educational level	Up to incomplete secondary education	191 (85%)	35 (15%)	0.42
	Graduated secondary education or attended higher education	67 (81%)	16 (19%)	
Family income	≥ 3 times minimum salary	27 (79%)	07 (21%)	0.49
	< 3 times minimum salary	231 (84%)	44 (16%)	
Time since diagnosis of diabetes mellitus	< 10 years	84 (88%)	12 (12%)	0.19
	≥ 10 years	173 (82%)	39 (18%)	

## DISCUSSION

The ABI is an important tool for diagnosis and estimation of prognosis of PAOD. However, in patients with diabetes it can be falsely elevated or excessively high because of calcification of the arterial tunica media, which can impede or stop arterial flow while the pressure cuff is being inflated.

One of the challenges with using ABI as a prognostic factor of atherosclerotic and cardiovascular disease is the difficulty in defining the ideal cutoff point for high ABI that is indicative of medial artery calcification, creating resistance to compression by the cuff. The literature also suggests that, for diagnostic purposes, the safest upper cutoff value is 1.3,^2^^-^ 4 ^,^ 6 ^,^ 7 ^,^ 14 since from this point on it is considered possible to confirm, and not just suggest, that patients have medial artery calcification.

The lower ABI cutoff most frequently used as a prognostic test for mortality from cardiovascular disease, indicated as a risk factor, is ABI < 0.9.^4^^,^ 5 However, with relation to high ABI, one international study has shown that elevated values greater than 1.4 can also represent an increased risk of cardiovascular mortality.^4^ Brazilian publications indicate a value of 1.3 as a cutoff point for high ABI, above which it can be considered a cardiovascular risk factor.^15^^,^ 16 Some authors suggest that pathological processes have onset at around 1.15, and so cardiovascular risk may become relevant from this point onwards.^3^^,^ 4 ^,^ 8

The value used as the cutoff point in this study is that proposed by the majority of studies; i.e. 1.3.^2^^,^ 3 The frequency of high ABI (≥ 1.3) in our sample comprising only patients with type 2 DM, was 16.5%, which is lower than the rate reported in the international literature, which varies around 21%.^4^^,^ 5 Moon et al.^13^ conducted a study to evaluate the presence of medial arterial calcification using radiographic examinations and found the abnormality in 21.2% of diabetics and just 5% of people who were not diabetic. A Brazilian study that measured the ABI of 73 diabetic patients with a mean age of 55.7 years found that 9.6% of them had ABI > 1.4.^17^

With regard to the age of the sample, the majority of patients were over 56 years of age and had been sent to a referral clinical for treatment of diabetes with complications from the disease. We did not detect an association between high ABI and age dichotomized around a 56-years-of-age cutoff; the younger subset did exhibit a lower frequency of high ABI, but the difference was not statistically significant. Since the aging process predisposes to calcification of the tunica media and other authors have detected an association between age and this condition detected radiologically,^13^ we stratified the sample into three distinct age groups. However, we still did not detect a significant association between the three different age groups and ABI ≥ 1.3. We believe that the failure to detect an association between age and excessively high ABI in our study may be attributable both to the sample size and to the fact that more than 40% of the patients were less than 56 years old, so they were probably too young for the process of arterial calcification to have initiated.

Another association tested was between the time since diagnosis of type 2 DM and high ABI. It would be expected that patients with longer disease duration would exhibit a higher frequency of high ABI. Patients diagnosed with diabetes more than 10 years previously exhibited a higher prevalence of high ABI, but without statistical significance. We stratified the sample into three subsets in an attempt to reveal this association, but although the frequency of excessively high ABI was slightly higher with longer duration of diabetes, the difference was still not statistically significant. We consider that it may be difficult for many of our patients to state with certainty the duration of their diabetes. Many Brazilians have difficulty accessing health services and obtaining follow-up and so the point at which a diagnosis of diabetes is made is imprecise and subjective. The disease is sometimes diagnosed at the time that complications emerge and there is also the difficulty with determining the exact time of diabetes. It is therefore difficult to make an exact assessment of these times.

The analyses of the other sociodemographic data (family income and educational level) also failed to demonstrate statistically significant associations with excessively high ABI. We did not find evidence in the literature demonstrating that these factors modify the frequency of high ABI, but we nevertheless studied these factors because we are aware of the difficulties that patients have with locating information and accessing adequate follow-up, which facilitate control and lead to a lower rate of complications from the disease.

In our analysis, we observed a slightly higher frequency of high ABI among females, but there was no significant difference between the sexes. Moon et al.^13^ found an association between male sex and calcification of the tunica media diagnosed with a radiographic examination. This finding might be because of the fact that the majority of our sample were women. One Brazilian study showed that, in patients with critical ischemia, females had a higher prevalence of DM and more extensive PAOD, with a lower number of opaque arteries visible in angiographies of the leg.^18^

Our study discusses a subject that is important in management of diabetic patients and assesses the socioeconomic characteristics of this group, having used an easily conducted and low-cost examination (ABI) with potential benefits for this population. It also reaffirms the need for measurement of ABI as part of clinical assessment of diabetic patients, since in addition to its diagnostic importance, public health policies can be based on well-founded data on this group of patients, who are at increased cardiovascular risk. Our study is subject to the limitations inherent to a cross-sectional study and it did not assess the patients in the sample for other important factors associated with DM at the same time or for comorbidities or progression of peripheral arterial disease or of cardiovascular disease.

## CONCLUSIONS

We conclude that the frequency of high ABI (≥ 1.3) among type 2 diabetic patients was 16.5%. In our sample, we did not find correlations between sex, age, time since DM diagnosis, educational level or family income and the frequency of high ABI.
